# Pediatric inflammatory multisystem syndrome induced Panuveitis associated with SARS-CoV- 2 infection: What the Ophthalmologists need to know

**DOI:** 10.22336/rjo.2022.39

**Published:** 2022

**Authors:** Corina Ioana Merticariu, Mircea Merticariu, Claudina Cobzariu, Mara Mădălina Mihai, Mihaela Sorina Dragomir

**Affiliations:** * Department of Ophthalmology, “Dr. Victor Gomoiu” Children’s Hospital, Bucharest, Romania; ** Department of Urology, CF2 Hospital, Bucharest, Romania; *** “Titu Maiorescu” University of Medicine, Bucharest, Romania; **** Department of Infectious Diseases, “Dr. Victor Gomoiu” Children’s Hospital, Bucharest, Romania; ***** “Carol Davila” University of Medicine and Pharmacy, Bucharest, Romania; ****** Department of Dermatology, “Elias” University Emergency Hospital, Bucharest, Romania

**Keywords:** Panuveitis, Optic nerve edema, SARS-CoV-2, pediatric inflammatory multisystem syndrome temporally associated with SARS-CoV-2 (PIMS-TS), multisystem inflammatory syndrome in children (MIS-C)

## Abstract

The diagnosis of bilateral panuveitis was made in a 9-year-old girl who was referred to our hospital for blurred vision accompanied by periorbital and abdominal pain. Endothelial dusting, vitreous haze and optic nerve edema were deemed as signs of involvement of all segments of the eye.

The bloodwork results were suggestive of infectious uveitis, with elevated inflammatory markers and the patient was treated with IV antibiotics. Cerebral-CT was normal, screening for common infectious causes of uveitis and cultures were negative. There was no history of autoimmune disease, and autoimmune antibody tests were negative. Pediatric inflammatory multisystem syndrome induced panuveitis, secondary to SARS-CoV-2 (PIMS), was suspected by the infectious disease consultant. The syndrome commonly affects school-age children and represents a generalized inflammatory response in the body that appears about one month after the initial infection with the SARS-CoV-2 virus. Initial symptoms include fever, abdominal pain, eye redness, rashes, dizziness, accompanied by laboratory evidence of inflammation unexplained by any other plausible cause. The patient’s coronavirus IgG titer was positive, while the RT-PCR for SARS-CoV-2 virus, taken from the nasopharyngeal swab, was negative. As all the other investigations turned out negative, COVID-19 was the only presumptive cause for the pediatric multisystem inflammatory syndrome temporally associated with SARS-CoV-2 (PIMS-TS). A diagnosis of probable COVID-19 induced uveitis was made and the patient started IV Dexamethasone, followed by oral steroids that were gradually tapered and made a full recovery.

The aim of this report was to shed light and enrich the scarce literature available on Uveitis as a sign of pediatric inflammatory syndrome following COVID-19 infection.

**Abbreviations:** ACE2 = Angiotensin converting enzyme 2, ANA = Antinuclear antibodies, c-ANCA, p-ANCA = Cytoplasmic and perinuclear anti-neutrophil cytoplasm antibodies, BCVA = Best corrected visual acuity, CMV = Cytomegalovirus, COVID-19 = coronavirus disease 2019, CRE = Carbapenem-resistant Enterobacteriaceae, CRP = C-Reactive Protein, EBV = Epstein Barr virus, ESBL = Extended spectrum beta-lactamase, ESR = Erythrocyte Sedimentation Rate, FCoV = Feline coronavirus, MDR = Multidrug resistant, MRSA = methicillin-resistant Staphylococcus aureus, MHV = mouse hepatitis virus, MIS-C = multisystem inflammatory syndrome in children, NSAID = Nonsteroidal anti-inflammatory drug, NT pro BNP = precursor natriuretic brain peptide, PIMS-TS = Pediatric inflammatory multisystem syndrome temporally associated with SARS-CoV-2, RNFL = Retinal nerve fiber layer, SARS CoV-2 = severe acute respiratory syndrome coronavirus 2, SD-OCT = Spectral domain optical coherence tomography, VRE = Vancomycin-resistant Enterococci

## Introduction

Covid-19 proved to be a multiorgan disease, with pulmonary and extrapulmonary involvement alike. Recent reports suggest that SARS-CoV-2 induces conjunctivitis and other thrombotic complications like central retinal vein occlusion [**1**]. Multiple organs have been affected by the SARS-CoV-2 virus, but ophthalmologic and neuro-ophthalmologic symptoms due to anterior or posterior Uveitis, Intracranial hypertension, or Optic Neuritis, have rarely been reported [**2**-**4**].

ACE2 receptors have been identified in the choroid, neurons, and vascular endothelium [**2**]. Various animal models established that COVID ocular infections can appear in mice and cats. Feline Cov (FCoV) and murine CoV mouse hepatitis virus (MHV) can produce an eye inflammatory disease that affects various segments of the eye [**2**,**3**]. These include vasculitis, retinitis, choroiditis and uveitis and optic neuritis, with complications that can lead to retinal atrophy and retinal detachment. This evidence suggests that the SARS-CoV-2 virus has a neuro tropism, as well as a tropism for the vascular endothelium tropism of SARS-CoV-2, supporting the assumption that it can cause the panuveitis [**4**-**8**]. 

The CDC has defined MIS-C associated with COVID-19: (a) Individual < 21 years of age with fever, laboratory evidence of inflammation, and clinically severe illness requiring hospitalization, with multisystem (≥ 2) organ involvement (cardiac, renal, respiratory, hematologic, gastrointestinal, dermatologic, or neurologic); (b) No alternative plausible diagnosis; (c) Positive current or recent SARS-CoV-2 infection quantified by RT-PCR, serology, or antigen test; alternatively, COVID-19 exposure within 4 weeks prior to symptoms. Fever is defined as temperature above 38.0°C for more than 24 hours or a subjective fever lasting longer than 24 hours. Laboratory evidence of inflammation is defined as: elevated erythrocyte sedimentation rate, fibrinogen, C-reactive protein, procalcitonin, D-dimer, interleukin-6 (IL-6, ferritin, and lactate dehydrogenase [**7**]. Pediatric patients are required to meet all three of the above-mentioned criteria. In this report, we described the clinical profile and treatment outcome of a patient who probably developed uveitis secondary to pediatric inflammatory multisystem syndrome temporally associated with SARS-CoV-2 (PIMS-TS).

## Case presentation

A 9-year-old girl presented to the Emergency Department of her hometown for abdominal pain accompanied by redness around the eye. She was diagnosed with right renal colic and conjunctivitis and was treated as an outpatient with systemic NSAIDS and a topical antibiotic. Two days after she developed pain around the eyes, she went to the local ophthalmologist who diagnosed her with anterior uveitis and started topical steroid and antibiotic treatment with a combination eye drop that contained Tobramycin and Dexamethasone. Five days after, she felt unwell with episodes of headache, shivers, and intense perspiration. Temperature was below 38oC, and sinus X-ray was normal, so a diagnosis of rhinitis was made and the patient started oral antibiotics with a combination of Amoxicillin and Clavulanic Acid. Several days after, she developed episodes of blurred vision, ocular pain and sleepiness and returned for an eye exam. Optic nerve edema was diagnosed, and she was referred to “V. Gomoiu” Children’s Hospital. During repeated consults, no Covid related respiratory symptoms were reported, and repeated SARS-CoV-2 antigen tests were negative.

At the initial examination after hospitalization, her best-corrected visual acuity (BCVA) was 0.6 in the right eye and 1 in the left eye. Color vision and confrontational fields were normal. Slit‐lamp examination showed conjunctival hyperemia, with perikeratic reaction, inferior corneal edema, endothelial dusting, and pigment dispersion on the anterior crystalloid in the right eye with a sluggish pupil that reacted poorly to light. Vitreous haze was observed, the optic disc was hyperemic and moderately elevated, with blurry margins. No posterior pole vascular sheathing was observed, and no other retinal peripheral lesions were detected. The left eye had a quiet anterior segment and a clear vitreous, normal retina and optic nerve. Intraocular pressure measured by non-contact tonometry was 9 mmHg in the right eye and 12 mmHg in the left eye. 

**Fig. 1:**
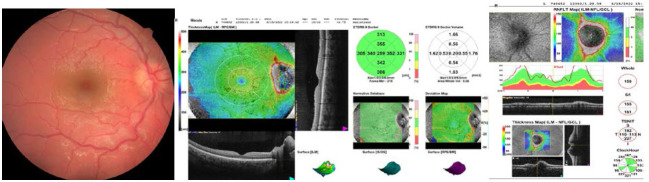
Fundus photo showing Frisen grade 2-3 optic nerve edema in the right eye with a circumferential halo of edema, disc hyperemia, elevation of the nasal border of the disc, partial obscuration of the inferior nasal branch of the central retinal vein leaving the disc, obliteration of the optic disc cup. OCT showing increased RNFL in all quadrants with a normal macula

Spectral domain Optical Coherence Tomography (SD-OCT) (Nidek, USA) confirmed the suspicion and proved a moderate edema in the retinal nerve fiber layer in the right eye (**Fig. 1**). 

The patient’s blood work showed moderate leukocytosis, thrombocytosis, and systemic inflammatory syndrome (ESR 110 mm/ h, CRP 95.84 mg/ L, Fibrinogen 470 mg/ dL, Procalcitonin 0.038 mcg/ L). Measured temperature was 37.8°C. The laboratory findings are shown in **Table 1**.

**Table 1:** Comparative bloodwork results during hospitalization

Laboratory test	Initial Value 15.03	18.03	On discharge 22.03	Normal values
RBC	4.22	4.58	5.2	4.5-5.9 x 10^9/ L
WBC	13.9	11.12	10.25	4.5-13.5 x 10^9/ L
NEUT	10.64	8.19	11.46	1.6-7.3 x 10^9/ L
PLT	538	581	612	135-475 x 10^9/ L
CRP	95.84	53.49	5.14	< 5 mg/ L
ESR	110	120	70	0-15 mm/ h
Fibrinogen	470.098	535.7		189-475 mg/ dL
Pro-Calcitonin	0.038	< 0.02	< 0.02	< 0.02 mcg/ L
IL6	No reactive			
C3		187.09		90-180 mg/ mL
D-dimer		309.472	160.065	0-255 ng/ mL
NT-pro BNP		164.5	81.75	0-125.2
LDH			125.06	120-300
Anti CMV IgM, IgG	< 5			Neg < 18 UI/ mL
Anti EBV IgM	18.8			Neg < 20 UI/ mL
Toxoplasma Gondi IgM	0.17			Neg < 0.55 UI/ mL
Toxoplasma Gondi IgG	1.3			Neg < 9 UI/ mL
Toxocara Canis IgG	0.8			Neg < 0.9 UI/ mL
RF	8.54			0-14 UI/ mL
ANA	0.2			< 1 UI/ mL
c-ANCA	2.9			< 7 UI/ mL
p-ANCA	0.4			< 5 UI/ mL
SARS-CoV-2 IgG	363			Neg < 5.5 AU/ ml
SARS-CoV-2 IgM	1.633			Neg < 3.0 AU/ ml

**Fig. 2:**
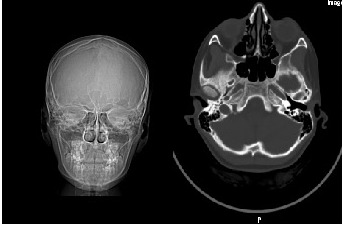
Cerebral CT scan with coronal and axial views showing normal bone structure, with no fluid accumulation within sinuses, no periorbital or cerebral inflammation

Infectious uveitis due to a local source was suspected, so treatment with IV antibiotics was started (Cefort 1 g every 12 hours for 7 days), accompanied by topical treatment with hourly steroid and antibiotic drops (Tobradex), and mydriatics three times per day. She had a normal cerebral CT scan (**Fig. 2**) and the Pediatric consultation and ENT exams were within normal limits. The rest of the Blood investigations, QuantiFERON, Toxocara Canis IgM, IgG, Toxoplasma Gondi, CMV, EBV were negative. Pharyngeal exudate culture was absent for Beta-hemolytic streptococcus, screening for carrying multi-resistant strains - nasal swab, rectal swab, skin swab, axillary and inguinal swab were absent for MDR+ (MRSA, ESBL, CRE, VRE). levels, ANA, C-ANCA, p- ANCA antibodies and ACE levels were normal. The patient had no history of autoimmune disease, no joint pain, no skin rashes, and no positive criteria for Bechet’s disease.

Two days after, while on antibiotic therapy, the patient’s left eye developed conjunctival hyperemia and periorbital pain. On fundus examination, the optic disc was hyperemic, and the optic nerve margins were slightly blurry at the superior and inferior poles. By this time, her CRP had decreased significantly to 53.49 mg/ L, but the ESR and fibrinogen had increased slightly, despite the IV antibiotic treatment. The NT- pro BNP, Complement C3, and D-dimer were elevated (NT-pro BNP 164.5, C3 187.09, D-dimer 309.472). 

**Fig. 3:**
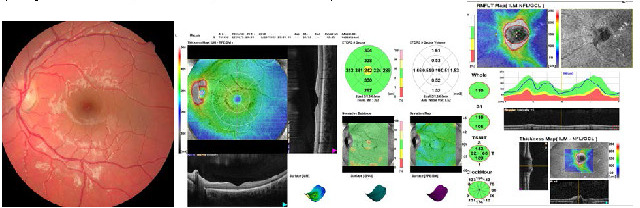
Fundus photo and OCT showing Frisen grade 1 optic nerve edema in a C-shaped halo in the left eye with blurred margins of the optic nerve and RNFL thickness at the upper limit of the normal range in the superior, inferior, and nasal quadrants

The patient had a SARS-CoV-2 IgG test, three weeks after her abdominal pain and conjunctivitis, and the test came back positive. Pulmonary X-ray showed peri-bronchial thickening and increased interstitial pattern in the lower fields, a common finding in COVID-19 patients (**Fig. 3**) [**9**,**10**]. On further discussion, the patient recalled that about 6 weeks prior to the development of her symptoms, while on school break, she visited family members who later developed anosmia. The symptoms were mild and transitory, and they were not tested for COVID-19. However, the patient was asymptomatic and had a negative antigen test when she returned to school, 4 days after meeting with the family. Therefore, at no time was she positive for SARS-CoV-2 infection.

**Fig. 4:**
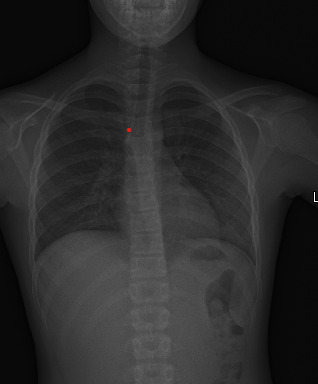
Antero-Posterior Chest X-ray showing bilateral subtle interstitial reticular pattern in the peripheral inferior pulmonary lobes and slightly increased peribronchovascular markings

According to scientific literature, common signs and symptoms of PIMS-TS are [**11**,**12**]: generalized hyperinflammation associated with non-specific gastrointestinal complaints, such as abdominal pain, vomiting or diarrhea, cardiac dysfunction, mucocutaneous signs (e.g., rash, conjunctivitis) and neurological symptoms such as headache. Kawasaki disease was considered as a differential, but the criteria were not met, as there was no fever lasting more than 5 days, no oral mucous, no polymorphous rash, no cervical lymph nodes and no peripheral signs associated to the bulbar conjunctival injection. 

Based on these findings, the diagnosis of panuveitis secondary to PIMS was made after excluding all other possible infectious and noninfectious causes. Due to the inflammatory response and blood work tests, IV dexamethasone was started at a moderate dose (0.4 mg/ kg/ day). Abdominal ultrasound showed: a normal liver, pancreas, kidneys and uterus, no liquid in the Douglas space. Cardiologic consult showed minor mitral regurgitation with small VMA prolapse and normal pericardium. Surgical consult was normal.

On discharge, the inflammatory syndrome had almost completely recovered. The patient could see 20/ 20 with each eye, color vision was normal with normal visual fields and a normal anterior segment examination. The optic disc was slightly hyperemic in both eyes, with blurry nasal margin in the right eye, and normal contour in the left eye. She had normal macula and peripheral retina in both eyes. Systemic and local steroid therapy was tapered progressively over one week.

## Discussion

COVID-19 illness is a progressing disorder with multiple manifestations in different organs reported so far [**13**,**14**]. We reported a rare case of PIMS-TS secondary uveitis, which was initially unilateral, and quickly became bilateral, and are considering it as a case of probable PIMS-TS induced uveitis based on the clinical findings, temporal association with SARS-CoV-2 exposure, and after ruling out other etiologies.

PIMS-TS cases usually become clinically apparent about four to six weeks after the number of COVID-19 cases peak in a certain area [**6**,**15**]. Even though the exact mechanism behind it is unknown, PIMS-TS probably represents a postinfectious cytokine-mediated hyperinflammatory process, triggered by COVID-19 infection [**13**-**15**]. Although the exact chain of events leading to the presumed post-viral hyperinflammation of PIMS-TS has not been established so far, the temporal link between SARS-CoV-2 exposure and the development of symptoms in patients with PIMS-TS make it highly plausible for SARS-CoV-2 to be the direct cause for PIMS-TS [**10**,**13**,**15**].

Some of the most common signs in children with PIMS-TS include abdominal pain (66.5%), vomiting (64.3%), rash (55.6%), diarrhea (53.7%), and conjunctival hyperemia (53.6%) [**10**,**16**-**20**]. 

The current case report highlighted one of the ophthalmologic complications of COVID-19. According to our literature research, this could be the first clinical report bilateral panuveitis induced by PIMS-TS [**2**-**6**,**9**,**10**,**20**]. 

There are scientific papers that reported conjunctivitis, uveitis, and optic neuritis in COVID-19 patients, but few studies mentioned the risk of vision loss due to COVID-19. Complaints associated with SARS-CoV-2 infection include pain, redness, and photophobia, suggesting probable eye surface disease. We found no papers that analyzed the possibility that the patient could have had a paucisymptomatic SARS-CoV-2 initial infection, and that the eye involvement eventually led to the diagnosis of PIMS-TS [**21**-**26**].

 We detailed the steps we took to make the diagnosis of panuveitis secondary to SARS-CoV-2 viral infection in pediatric patient. The patient’s Covid antibodies were positive while the RT-PCR for SARS-CoV-2 virus was negative. Clinical, radiological and bloodwork investigations suggested the possibility of pediatric COVID-19 inflammatory syndrome (PIMS-TS) with secondary uveitis. What is interesting is that temperature measured was never above 38oC, but the patient had repeated episodes of shivers followed by intense perspiration at the same time she developed her abdominal pain, making it plausible to assume that she had fever even if it was never measured with a thermometer. Moreso, the initial conjunctivitis that preceded the uveitis could have been a sign of acute infection, spreading through the conjunctiva [**27**-**29**]. 

 The patient showed remarkable recovery following steroid therapy. Other case reports advocate the early use of corticosteroids, demonstrating that a short course of a moderate dose of steroids may speed recovery from COVID-19 pulmonary and extrapulmonary disease [**1**,**30**-**32**]. 

Current guidelines for autoimmune diseases recommend the use of the lowest possible dose of steroid medication, in the absence of COVID-19 [**33**]. On the other hand, the World Health Organization (WHO) recommends the avoidance of systemic corticosteroids in severe cases of COVID-19 with pulmonary and extrapulmonary involvement [**34**]. However, a clinical trial from UK demonstrated that low levels of dexamethasone can be beneficial in COVID-19 patients and can reduce the mortality. Still, different doctors from different settings have controversial views regarding the use of steroid therapy in COVID-19 cases and a consensus between experts does not seem within reach [**30**-**35**]. 

This case report highlighted the importance of ocular symptoms, which can be easily overlooked if atypical cases of COVID-19 do not have concurrent systemic symptoms. There have been countless reports of ocular and neurological complications since the onset of the coronavirus pandemic [**2**,**20**-**24**,**28**]. The neuro-ophthalmological symptoms are considered to represent a separate entity of COVID-19 related manifestations, with multiple underlying mechanisms, such as the high affinity for ACE2 receptors, whose existence in the eye, nerves and vascular endothelium has already been demonstrated [**2**-**5**,**7**,**16**,**17**,**25**-**28**, **30**-**31**]. In this case, the panuveitis could be a feature of PIMS temporally associated with SARS-CoV-2 infection, but this remains a hypothesis and warrants further investigations and follow-up due to the risk of vision loss and the association with systemic involvement.

Studies demonstrated that, compared with children with severe COVID-19, children with PIMS-TS had higher levels of IgG antibodies that neutralize SARS-CoV-2 more effectively. The reason behind this finding may be due to a longer time lapse from the onset of infection in children with PIMS-TS compared to children with severe COVID-19, but this is difficult to prove since most patients do not recall a specific moment of exposure or symptoms compatible with acute disease [**13**,**14**,**19**]. 

As most of young children with COVID-19 have a wide variety of generally mild to moderate manifestations that are not specific enough to require SARS-CoV-2 testing, and adolescents may be completely asymptomatic, the challenge is to recognize COVID-19 changes and properly treat them.

## Conclusion

Through this case, we advocate the importance of taking a detailed medical history, looking at the patient as a whole and understanding the mechanisms underlying organ involvement by the cytokine storm, since it can rapidly lead to multi-organ inflammatory syndrome.

The ocular findings significantly improved after corticosteroid therapy. We support the early and appropriate use of corticosteroids in PIMS-TS cases, taking into consideration the age of the patient, the severity of the uveitis and the preexisting ocular and systemic comorbidities. More papers are necessary to adequately cover the wide array COVID-19 and PIMS-TS manifestations and to help health care providers in the detection of early cases. 

In conclusion, we cannot emphasize enough the importance of keeping an open mind regarding the inflammatory involvement during and after SARS-CoV-2 infection. All health care providers should have a strong suspicion of PIMS-TS uveitis when dealing with patients with a prior history of viral prodromal symptoms, including atypical manifestations such as conjunctivitis.

**Conflict of Interest statement**

The authors state no conflict of interest.

**Informed Consent and Human and Animal Rights statement**

Informed consent has been obtained from the individual included in this study.

**Authorization for the use of human subjects**

Ethical approval: The research related to human use complies with all the relevant national regulations, institutional policies, is in accordance with the tenets of the Helsinki Declaration, and has been approved by the review board of the Department of Ophthalmology of “Victor Gomoiu” Children’s Hospital, Bucharest, Romania.

**Acknowledgements**

None.

**Sources of Funding**

None.

**Disclosures**

None.
